# The glucose ketone index calculator: a simple tool to monitor therapeutic efficacy for metabolic management of brain cancer

**DOI:** 10.1186/s12986-015-0009-2

**Published:** 2015-03-11

**Authors:** Joshua J Meidenbauer, Purna Mukherjee, Thomas N Seyfried

**Affiliations:** Biology Department, Boston College, Chestnut Hill, MA 02467 USA

**Keywords:** Glucose, Beta-hydroxybutyrate, Calorie restriction, Metabolic therapy, Glioblastoma, Warburg effect, Ketogenic diet, Ketone bodies

## Abstract

**Background:**

Metabolic therapy using ketogenic diets (KD) is emerging as an alternative or complementary approach to the current standard of care for brain cancer management. This therapeutic strategy targets the aerobic fermentation of glucose (Warburg effect), which is the common metabolic malady of most cancers including brain tumors. The KD targets tumor energy metabolism by lowering blood glucose and elevating blood ketones (β-hydroxybutyrate). Brain tumor cells, unlike normal brain cells, cannot use ketone bodies effectively for energy when glucose becomes limiting. Although plasma levels of glucose and ketone bodies have been used separately to predict the therapeutic success of metabolic therapy, daily glucose levels can fluctuate widely in brain cancer patients. This can create difficulty in linking changes in blood glucose and ketones to efficacy of metabolic therapy.

**Methods:**

A program was developed (Glucose Ketone Index Calculator, GKIC) that tracks the ratio of blood glucose to ketones as a single value. We have termed this ratio the Glucose Ketone Index (GKI).

**Results:**

The GKIC was used to compute the GKI for data published on blood glucose and ketone levels in humans and mice with brain tumors. The results showed a clear relationship between the GKI and therapeutic efficacy using ketogenic diets and calorie restriction.

**Conclusions:**

The GKIC is a simple tool that can help monitor the efficacy of metabolic therapy in preclinical animal models and in clinical trials for malignant brain cancer and possibly other cancers that express aerobic fermentation.

**Electronic supplementary material:**

The online version of this article (doi:10.1186/s12986-015-0009-2) contains supplementary material, which is available to authorized users.

## Introduction

Dietary therapy using ketogenic diets is emerging as an alternative or complementary approach to the current standard of care for brain cancer management. Prognosis remains poor for malignant gliomas in both children and adults [1-5]. Although genetic heterogeneity is extensive in malignant gliomas [6-8], the Warburg effect (aerobic fermentation of glucose) is a common metabolic malady expressed in nearly all neoplastic cells of these and other malignant tumors [9-11]. Aerobic fermentation (Warburg effect) is necessary to compensate for the insufficiency of mitochondrial oxidative phosphorylation in the cells of most tumors [9,12-14]. Mitochondrial structure and function is abnormal in malignant gliomas from both mice and humans [15-19]. Normal brain cells gradually transition from the metabolism of glucose to the metabolism of ketone bodies (primarily β-hydroxybutyrate and acetoacetate) for energy when circulating glucose levels become limiting [20,21]. Ketone bodies are derived from fatty acids in the liver and are produced to compensate for glucose depletion during periods of food restriction [20]. Ketone bodies bypass the glycolytic pathway in the cytoplasm and are metabolized directly to acetyl CoA in the mitochondria [22]. Tumor cells are less capable than normal cells in metabolizing ketone bodies for energy due to their mitochondrial defects [2,12,23].

**Figure 1:**
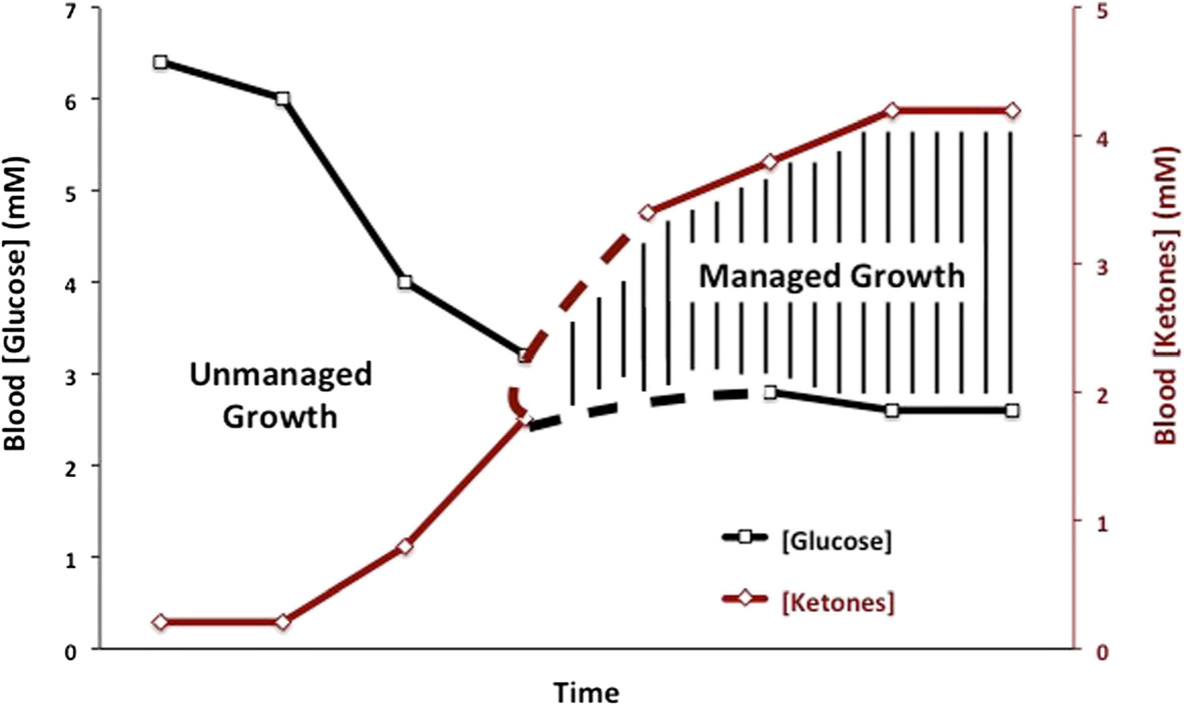
**Relationship of plasma glucose and ketone body levels to brain cancer management.** The glucose and ketone (β-­OHB) values are within normal physiological ranges under fasting conditions in humans. We refer to this state as the zone of metabolic management. As blood glucose falls and blood ketones rise, an individual is predicted to reach the zone of metabolic management. Tumor progression is predicted to be slower within the metabolic target zone than outside of the zone. This can be tracked utilizing the Glucose Ketone Index. The dashed lines signify the variability that could exist among individuals in reaching a GKI associated with therapeutic efficacy.

Therapies that can lower glucose and elevate ketone bodies will place more energy stress on the tumor cells than on the normal brain cells [12,24]. This therapeutic strategy is illustrated conceptually in Figure 1, as we previously described [25]. However, daily activities and emotional stress can cause blood glucose levels to vary making it difficult for some people to enter the predicted zone of metabolic management [26]. A more stable measure of systemic energy metabolism is therefore needed to predict metabolic management of tumor growth. The ratio of blood glucose to blood ketone bodies β-hydroxybutyrate (β-OHB) is a clinical biomarker that could provide a better indication of metabolic management than could measurement of either blood glucose or ketone body levels alone.

## Methods

The ‘Glucose Ketone Index’ (GKI) was created to track the zone of metabolic management for brain tumor management. The GKI is a biomarker that refers to the molar ratio of circulating glucose over β-OHB, which is the major circulating ketone body. A mathematical tool called the Glucose Ketone Index Calculator (Additional file 1) was developed that can calculate the GKI and monitor changes in this parameter on a daily basis (Equation 1). The GKIC generates a single value that can assess the relationship of the major fermentable tumor fuel (glucose) to the non-fermentable fuel (ketone bodies). Because many commercial blood glucose monitors give outputs in mg/dL, rather than millimolar (mM), the GKIC converts the units to millimolar. Included in the program is a unit converter for both glucose and ketones (β-OHB), which can convert glucose and ketone values from mg/dL to mM and from mM to mg/dL (Equations 2, 3, 4, 5). The molecular weights used for calculations in the GKIC are 180.16 g/mol for glucose and 104.1 g/mol for β-OHB, which is the major circulating ketone body measured in most commercial testing kits. The unit converter allows for compatibility for a variety of glucose and ketone testing monitors.

1 $$ \left[\mathrm{Glucose}\ \mathrm{Ketone}\ \mathrm{Index}\right]=\frac{\raisebox{1ex}{$\left[\mathrm{Glucose}\ \left(\mathrm{m}\mathrm{g}/\mathrm{dL}\right)\right]$}\!\left/ \!\raisebox{-1ex}{$18.016\ \left(\mathrm{g}*\frac{\mathrm{dL}}{\mathrm{mol}}\right)$}\right.}{\left[\mathrm{Ketone}\ \left(\mathrm{m}\mathrm{M}\right)\right]} $$

2 $$ \left[\mathrm{Glucose}\ \left(\mathrm{m}\mathrm{g}/\mathrm{dL}\right)\right]=\left[\mathrm{Glucose}\ \left(\mathrm{m}\mathrm{M}\right)\right] \times 18.016\ \left(\mathrm{g}*\frac{\mathrm{dL}}{\mathrm{mol}}\right) $$

3 $$ \left[\mathrm{Glucose}\ \left(\mathrm{m}\mathrm{M}\right)\right]=\frac{\left[\mathrm{Glucose}\ \left(\mathrm{m}\mathrm{g}/\mathrm{dL}\right)\right]}{18.016\ \left(\mathrm{g}*\frac{\mathrm{dL}}{\mathrm{mol}}\right)} $$

4 $$ \left[\mathrm{Ketone}\ \left(\mathrm{m}\mathrm{g}/\mathrm{dL}\right)\right]=\left[\mathrm{Ketone}\ \left(\mathrm{m}\mathrm{M}\right)\right] \times 10.41\ \left(\mathrm{g}*\frac{\mathrm{dl}}{\mathrm{mol}}\right) $$

5 $$ \left[\mathrm{Ketone}\ \left(\mathrm{m}\mathrm{M}\right)\right]=\frac{\left[\mathrm{Ketone}\ \left(\mathrm{m}\mathrm{g}/\mathrm{dL}\right)\right]}{10.41\ \left(\mathrm{g}*\frac{\mathrm{dl}}{\mathrm{mol}}\right)} $$

**Figure 2:**
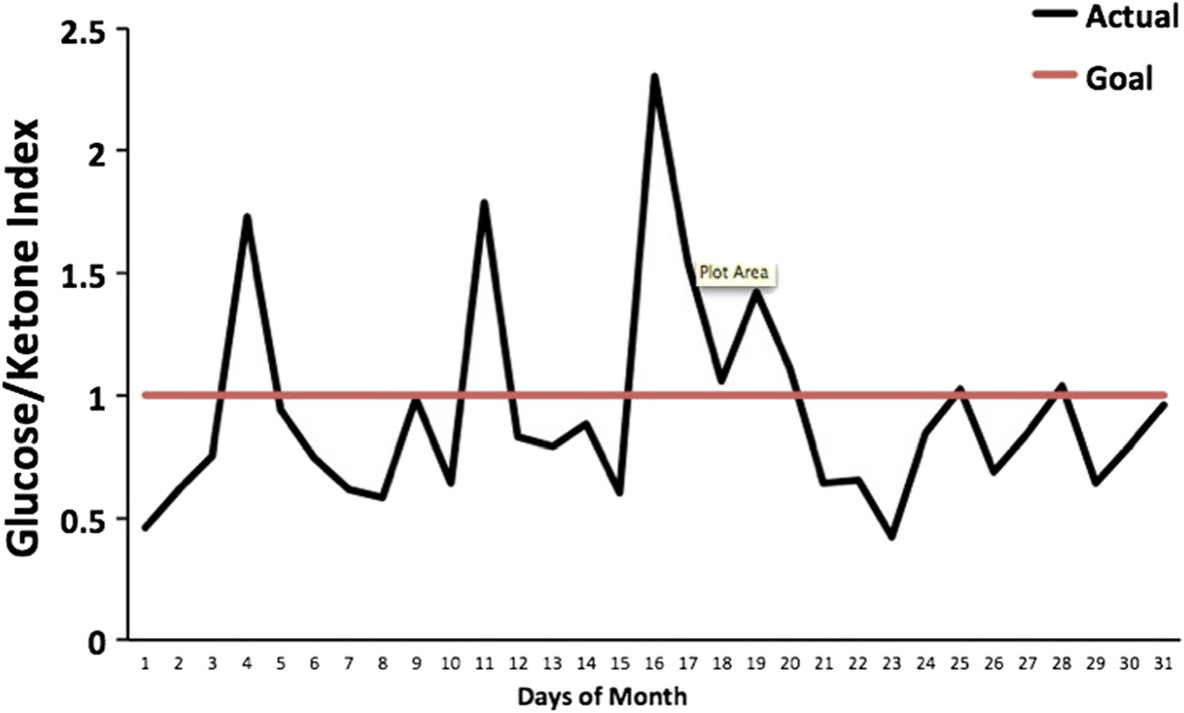
**The Glucose Ketone Index Calculator tracking an individual’s GKI.** The individual glucose and ketone values are displayed, along with the corresponding GKI values. The GKI values are plotted over the course of a month (black line), whereas the GKI target value (1.0) is plotted as a red line. We consider GKI values approaching 1.0 as potentially most therapeutic.

The GKIC can set a target GKI value to help track therapeutic status. Daily GKI values can be plotted to allow visual tracking of progress against an initial index value over monthly periods. Entrance into the zone of metabolic management would be seen as the GKI value falls below the set target value (as illustrated in Figure 2). Additionally, the GKIC can track the number of days that an individual falls within the predicted target zone.

## Results

The GKIC was used to estimate the GKI for humans and mice with brain tumors that were treated with either calorie restriction or ketogenic diets from five previously published reports (Table 1). The first clinical study evaluated two pediatric patients; one with an anaplastic astrocytoma, and another with a cerebellar astrocytoma [27]. Both individuals were placed on a ketogenic diet for eight weeks. During the 8-week treatment period, GKI dropped from about 27.5 to about 0.7 – 1.1 in the patients. The patient with the anaplastic astrocytoma, who did not have a response to prior chemotherapy, had a 21.7% reduction in fluorodeoxyglucose uptake at the tumor site (no chemotherapy during diet). The patient with the cerebellar astrocytoma received standard chemotherapy concomitant with the ketogenic diet. Fluorodeoxyglucose uptake at the tumor site in this patient was reduced by 21.8%. Quality of life was markedly improved in both children after initiation of the KD [27].

**Table 1:** **Low Glucose Ketone Index values are related to improved prognoses in humans and mice with brain tumors**

**Subjects**	**Tumor type**	**Diet**	**# of subjects**	**Days on diet**	**Glucose (mM)**	**Ketones** ^**f**^ **(mM)**	**Glucose Ketone Index**	**Prognosis**
Human^1^	Anaplastic Astrocytoma	KD-UR^a^	1	0	5.5	0.2	27.5	No response to standard chemotherapy
				56	5.0	4.6	1.1	FDG uptake at tumor site was decreased by 21.77%; tumor margins were unchanged
	Cerebellar Astrocytoma	KD-UR	1	0	5.5	0.2	27.5	Tumor resected and initiated on KD while under standard chemotherapy,after tumor was radiologically stable by CT
				56	4.0	5.5	0.7	FDG uptake at tumor site was decreased by 21.84%
								**Notes:** Both patients remained in remission after return to standard diet for 5 years (Subject 1) and 4 years (Subject 2),at time of publication
Human^2^	Glioblastoma	KD-R^b^	1	0	7.5	0.2^g^	37.5	Incomplete surgical resection of tumor; received chemotherapy and radiation therapy concurrent with diet
				21	3.5	2.5^g^	1.4	No evidence of tumor by MRI after concurrent therapy
								**Notes:** Patient stayed on low calorie diet for an additional 5 months; tumor recurrence 3 months after low-calorie diet suspension
Mouse^3^	Mouse CT-2A astrocytoma	SD-UR^c^	7	13	9.1	0.6	15.2	Tumor dry weight:55 ± 15mg^h^
	Syngenic (C57BL/6 J)	SD-R^d^	6	13	5.2	1.4	3.7	Tumor dry weight:7 ± 7 mg
		KD-UR	14	13	11.4	1.0	11.4	Tumor dry weight:70 ± 15 mg
		KD-R	6	13	5.7	1.3	4.4	Tumor dry weight:14 ± 8 mg
Mouse^4^	Mouse CT-2A astrocytoma	SD-UR	12-14	8	14.0	0.2	70.0	Tumor dry weight:95 ± 25mg^h^
	Syngenic (C57BL/6 J)	KD-UR	12-14	8	13.5	0.6	22.5	Tumor dry weight:90 ± 15 mg
		KD-R	12-14	8	8.0	1.8	4.4	Tumor dry weight:35 ± 5 mg
	Human U87-MG glioma	SD-UR	12-14	8	11.5	0.5	23.0	Tumor dry weight:60 ± 10mg^h^
	Xenograft (SCID)	KD-UR	12-14	8	11.5	1.2	9.6	Tumor dry weight:60 ± 7 mg
		KD-R	12-14	8	5.5	3.0	1.8	Tumor dry weight:37 ± 5 mg
Mouse^5^	Mouse GL261 glioma	SD-UR	19	13	10.0	0.2	50.0	Median survival time:23 days
	(C57BL/6-cBrd/cBrd/Cr)	KD-UR	19	13	8.9	1.4	6.4	Median survival time:28 days
		SD-UR + Rad^e^	11	13	9.7	0.3	32.3	Median survival time:41 days
		KD-UR + Rad	11	13	9.7	1.7	5.7	Median survival time:200 + days

^1^Nebeling *et al.*, 1995 [27]

^2^Zuccoli *et al.*, 2010 [28]

^3^Seyfried *et al.*, 2003 [29]

^4^Zhou *et al.*, 2007 [30]

^5^Abdelwahab *et al.*, 2012 [31]

^**a**^Ketogenic diet, unrestricted.

^b^Ketogenic diet, restricted.

^c^ Standard diet, unrestricted.

^d^ Standard diet, restricted.

^e^ Diet with radiation therapy.

^f^ Blood/plasma beta-hydroxybutyrate measurement.

^g^ Urinary ketones were measured.

^h^ Mean ± 95% Confidence Interval.

The second clinical study evaluated a 65-yr-old woman with glioblastoma multiforme [28]. The patient was placed on a calorie-restricted ketogenic diet (600 kcal/day) concomitant with standard chemotherapy and radiation, without dexamethasone, for eight weeks. The patient’s GKI decreased from 37.5 to 1.4 in the first three weeks of the diet. No discernible brain tumor tissue was detected with MRI in the patient at the end of eight weeks of the calorie restricted ketogenic diet. It is also important to mention that the patient was free of symptoms while she adhered to the KD. Tumor recurrence occurred 10 weeks after suspension of the ketogenic diet.

The third study, a preclinical mouse study, evaluated the effects of diets on an orthotopically implanted CT-2A syngeneic mouse astrocytoma in C57BL/6 J mice [29]. Mice were implanted with tumors and fed one of four diets for 13 days: 1) standard diet fed unrestricted, 2) calorie restricted standard diet, 3) ketogenic diet fed unrestricted, or 4) calorie restricted ketogenic diet. The mice fed a standard unrestricted diet and a ketogenic diet had rapid tumor growth after 13 days, and a GKI of 15.2 and 11.4, respectively. The group fed a calorie restricted standard diet had a significant decrease in tumor volume after 13 days, along with a GKI of 3.7. The group fed a calorie restricted ketogenic diet also had a significant decrease in tumor volume, along with a GKI of 4.4.

The fourth study evaluated the effects of diets on an orthotopically implanted CT-2A syngeneic mouse astrocytoma in C57BL/6 J mice and an orthotopically implanted human U87-MG human xenograft glioma in BALBc/6-severe combined immunodeficiency (SCID) mice [30]. Tumors were implanted and grown in the mice for three days prior to diet initiation. After three days, mice were maintained on one of three diets for 8 days: 1) standard diet fed unrestricted, 2) ketogenic diet fed unrestricted, or 3) calorie restricted ketogenic diet. Tumor weights at the end of 8 days were reduced only in the mice that were fed a calorie restricted diet and experienced a significant decrease in GKI. Groups of mice that did not have a reduction in tumor weight had GKI’s that ranged from 9.6 – 70.0. The groups of mice that had a reduction in tumor weight had GKI’s that ranged from 1.8 – 4.4.

The fifth study evaluated the effects of diet and radiation on mouse GL261 glioma implanted intracranially in albino C57BL/6 J mice [31]. The mice were implanted with tumors, and three days later they were placed on either a standard diet fed unrestricted or a ketogenic diet fed unrestricted. Mice were also assigned to groups that either received or did not receive concomitant radiation therapy. Without radiation, mice that were fed a ketogenic diet had a GKI of 6.4 and had a median survival of 28 days, compared to a GKI of 50.0 and median survival of 23 days for the standard diet group. With radiation, mice that were fed a ketogenic diet had a GKI of 5.7 and a median survival of 200+ days, compared to a GKI of 32.3 and median survival of 41 days for the standard diet group.

In addition to these studies, Table 2 shows a clear association of the GKI to the therapeutic action of calorie restriction against distal invasion, proliferation, and angiogenesis in the VM-M3 model of glioblastoma. The data for the GKI in Table 2 was computed from those mice that were measured for both glucose and ketones in comparison with the other biomarkers as previously described [32]. When viewed collectively, the results from the published reports show a clear relationship between the GKI and efficacy of metabolic therapy using either the KD or calorie restriction. Therapeutic efficacy of the KD or calorie restriction is greater with lower GKI values than with higher values. The results suggest that GKI levels that approach 1.0 are therapeutic for managing brain tumor growth. Further studies will be needed to determine those GKI values that can most accurately predict efficacy during metabolic therapy involving diet or procedures that lower glucose and elevate ketone bodies.

**Table 2:** **Linking the Glucose Ketone Index (GKI) to the therapeutic action of calorie restriction against distal invasion, proliferation, and angiogenesis in the VM-M3 model of glioblastoma**

**Treatment**	**Glucose (mM)**	**Ketone (mM)**	**GKI**	**Distal invasion (photons/sec)**	**Proliferation (Ki67 %)**	**Angiogenesis (vessels/hpf)**
**AL**	11.2 ± 0.6	0.7 ± 0.09	15.3 ± 0.9	14 ± 1.8	48 ± 1.2	15 ± 1.1
**CR**	8.3 ± 0.8	1.32 ± 0.1	6.5 ± 0.9	6 ± 0.9	34 ± 1.5	7 ± 0.72

AL, *ad libitum* feeding and CR is 60% food reduction for 10 days. Values are Mean ± SEM. 3-7 mice were evaluated in each group; hpf, high power field.

## Discussion

We present evidence showing that the GKI can predict success for brain cancer management in humans and mice using metabolic therapies that lower blood glucose and elevate blood ketone levels. Besides ketogenic diets, other dietary therapies, such as calorie restriction, low carbohydrate diets, and therapeutic fasting, can also lower blood glucose and elevate β-OHB levels and can have anti-tumor effects [24,33-38]. The GKIC was developed to more reliably and simply predict therapeutic management for brain cancer patients under these dietary states than could measurements of either blood glucose or ketones alone. The data presented in Tables 1 and 2 support this prediction. Although the GKI is simple in concept, it has not been used previously to gage success of various metabolic therapies based on inverse changes in glucose and ketone body metabolism.

As brain tumor cells are dependent on glucose for survival and cannot effectively use ketone bodies as an alternative fuel, a zone of metabolic management can be achieved under conditions of low glucose and elevated ketones. Ketone bodies also prevent neurological symptoms associated with hypoglycemia, such as neuroglycopenia, which allows blood glucose levels to be lowered even further [22,39]. Hence, ketone body metabolism can protect normal brain cells under conditions that target tumor cells [40]. The zone of metabolic management is considered the therapeutic state that places maximal metabolic stress on tumor cells while protecting the health and vitality of normal cells [41]. We have presented substantial data showing that the GKI is validated in several studies in mice. We feel that prospective validation of the GKIC will be obtained from future studies using ketogenic diet therapy in humans with brain cancer and possibly other cancers that cannot effectively metabolize β-OHB for energy, and depend upon glucose for survival.

The GKI can be useful in determining the success of dietary therapies that shift glucose- and lactate-based metabolism to ketone-based metabolism. As a shift toward ketone-based metabolism underscores the utility of many dietary therapies in treating metabolic diseases [41,42], the GKI can be used in determining the therapeutic success of shifting metabolism in individual patients. The GKI therefore can be used to study the effectiveness of dietary therapy in clinical trials of patients under a range of dietary conditions, with a composite primary endpoint consisting of lowering the subjects’ GKI. This will allow investigators to parse the effects of successful dietary intervention on disease outcome from unsuccessful dietary intervention.

Recent clinical studies assessing the effects of dietary therapy on brain cancer progression have not measured both blood glucose and ketone bodies throughout the study periods [43,44]. Future clinical studies that intend to assess the effect of dietary therapy on brain tumor progression should measure both blood glucose and ketone, as these markers are necessary to connect dietary therapy to therapeutic efficacy. Preclinical studies have demonstrated a clear linkage between GKI and therapeutic efficacy. The GKI will be an important biomarker to measure in future rigorously designed and powered clinical studies in order to demonstrate if there is a linkage between GKI and therapeutic efficacy, as the few case reports in the literature suggest.

The zone of metabolic management is likely entered with GKI values between 1 and 2 for humans. Optimal management is predicted for values approaching 1.0, and blood glucose and ketone values should be measured 2–3 hours postprandial, twice a day if possible. This will allow individuals to connect their dietary intake to changes in their GKI. As an example, Figure 2 uses the GKIC to track the GKI values of an individual on a ketogenic diet, with a target GKI of 1.0. When an individual’s GKI falls below the line denoting the target metabolic state, the zone of metabolic management is achieved. Further studies will be needed to establish the validity of the predicted zone of management.

It has not escaped our attention that the GKIC could have utility not only for managing brain cancer and possibly other cancers dependent on glucose and aerobic fermentation for survival, but also for managing other diseases or conditions where the ratio of glucose to ketone bodies could be therapeutic. Such diseases and conditions include Alzheimer’s disease, Parkinson’s disease, traumatic brain injury, chronic inflammatory disease, and epilepsy [41]. For example, the ketogenic diet has long been recognized as an effective therapeutic strategy for managing refractory seizures in children [45,46]. Therapeutic success in managing generalized idiopathic epilepsy in epileptic EL mice can also be seen when applying the GKI to the data presented on glucose and β-OHB [47]. Healthy individuals can utilize the GKIC to prevent diseases and disorder, and manage general wellness. Further studies will be needed to determine the utility of the GKIC for predicting therapeutic success in the metabolic management of disease.

## Additional file

Additional file 1:
**Instructions for calculating the GKI using a blood glucose and ketone monitor.**
